# Book Notices

**Published:** 1869-08

**Authors:** 


### Book Notices.

A Manual of Elementary Chemistry, Theoretical and Practical. By GEORGE FOWNES, F.R.S., late Professor of Practical Chemistry in the University College, London. From the tenth revised and corrected English edition. Edited by Robert Bridges, M.D., Professor of Chemistry in the Philadelphia College of Pharmacy. With 197 Illustrations. Philadelphia: Henry C. Lea. 1869.

This a new and carefully revised edition of a well-known text-book of practical chemistry. A number of important additions have been made, and it will continue, as heretofore, to hold the first rank as a text-book for students of medicine.

---

Monograph on Hemorrhagic Malarial Fever. Read to the Alabama State Medical Society, and republished from the New Orleans Journal of Medicine. By R. FRAZER MICHEL, M.D., of Montgomery, Alabama. 1869.

This is a very interesting monograph of only 19 pages, giving an account of the history, symptoms, pathology, and treatment of a very peculiar and fatal form of malarial fever, as it has prevailed in Alabama, Louisiana, and Texas during the last few years.

---

Carbolic Acid: Its Surgical and Therapeutical Uses. A paper read before the Cincinnati Academy of Medicine, June, 1869. By WM. B. DAVIS, M.D., of Cincinnati.

This is a paper of 13 printed pages, in which the author gives a very judicious summary of what is known of the actual surgical and therapeutical value of carbolic acid.

---

Treatment of Lachrymal Affections. By Prof. ARLT, Professor of Ophthalmology, at the University of Vienna. Translated by John F. Wightman, M.D. Philadelphia: Lindsay & Blakiston. 1869.

This is a monograph of 30 pages, in paper cover, but on excellent type and paper. It contains, in a brief compass and cheap form, the present state of our knowledge concerning a very troublesome class of affections.

